# Genetic tests obtainable through pharmacies: the good, the bad, and the ugly

**DOI:** 10.1186/1479-7364-7-17

**Published:** 2013-07-08

**Authors:** George P Patrinos, Darrol J Baker, Fahd Al-Mulla, Vasilis Vasiliou, David N Cooper

**Affiliations:** 1 Department of Pharmacy, School of Health Sciences, University of Patras, University Campus, Rion, Patras, 26504, Greece; 2 The Golden Helix Foundation, London, UK; 3 Faculty of Medicine, Department of Pathology, Kuwait University, Safat, Kuwait; 4 School of Pharmacy, University of Colorado, Denver, CO, 80202, USA; 5 Institute of Medical Genetics, School of Medicine, Cardiff University, Cardiff, Wales, UK

## Abstract

Genomic medicine seeks to exploit an individual’s genomic information in the context of guiding the clinical decision-making process. In the post-genomic era, a range of novel molecular genetic testing methodologies have emerged, allowing the genetic testing industry to grow at a very rapid pace. As a consequence, a considerable number of different private diagnostic testing laboratories now provide a wide variety of genetic testing services, often employing a direct-to-consumer (DTC) business model to identify mutations underlying (or associated with) common Mendelian disorders, to individualize drug response, to attempt to determine an individual’s risk of a multitude of complex (multifactorial) diseases, or even to determine a person’s identity. Recently, we have noted a novel trend in the provision of private molecular genetic testing services, namely saliva and buccal swab collection kits (for deoxyribonucleic acid (DNA) isolation) being offered for sale over the counter by pharmacies. This situation is somewhat different from the standard DTC genetic testing model, since pharmacists are healthcare professionals who are supposedly qualified to give appropriate advice to their clients. There are, however, a number of issues to be addressed in relation to the marketing of DNA collection kits for genetic testing through pharmacies, namely a requirement for regulatory clearance, the comparative lack of appropriate genetics education of the healthcare professionals involved, and most importantly, the lack of awareness on the part of both the patients and the general public with respect to the potential benefits or otherwise of the various types of genetic test offered, which may result in confusion as to which test could be beneficial in their own particular case. We believe that some form of genetic counseling should ideally be integrated into, and made inseparable from, the genetic testing process, while pharmacists should be obliged to receive some basic training about the genetic tests that they offer for sale.

## 

Genomic or personalized medicine seeks to exploit an individual’s genetic or genomic information in the context of guiding the clinical decision-making process [1]. Examination of a patient’s genome sequence can allow physicians to estimate disease risk and to individualize treatment modalities. At the same time, the molecular basis of a growing number of disease states has been elucidated and can now be readily identified by reference to specific genotypes and/or gene expression patterns. This has potentiated the risk stratification of patients for a wide variety of genetic disorders on a genome-wide basis [2]. We are now entering an age in which individualized health care is fast becoming a reality through consideration of each person’s unique genomic profile alongside their clinical profile. This should give rise to unprecedented opportunities in the context of inherited disease, not only in terms of optimizing preventive medicine strategies and customizing patient care but also with a view to personalizing conventional therapeutic interventions. Such interventions could potentially be made at an early stage in the onset of a genetic disorder or even pre-symptomatically [2]. Mutation-specific targeted intervention is already a reality in some cancer treatments [3].

At the same time, molecular genetic testing technology has evolved such that low- and medium-throughput methods have been largely replaced by high-throughput genome-wide microarray-based assays, an important advance which has allowed the genetic testing industry to grow at a very rapid pace [4]. Indeed, a considerable number of different private diagnostic testing laboratories now provide a wide variety of genetic testing services and over 2,000 specific genetic tests, often employing a direct-to-consumer (DTC) business model [4]. Genetic laboratories currently offer DTC antenatal carrier testing and/or pre-pregnancy family planning tests, which have been designed to identify mutations underlying common Mendelian disorders such as cystic fibrosis and β-thalassemia. However, in parallel, tests that purport to assess a person’s risk of a multitude of complex (multifactorial) diseases including heart/cardiovascular disease, hypertension, diabetes, osteoporosis, and lung and breast cancer are also available. In practice, despite the extravagant claims often made on behalf of the tests being offered, our current level of scientific understanding is usually insufficient to accurately assess the risk of common diseases such as cancer, diabetes, and heart/cardiovascular disease; even though data on genetic markers can be readily obtained and accurately ascertained, we are as yet unable to interpret them with any degree of accuracy or reliability [5-8] (see also http://www.gao.gov/new.items/d10847t.pdf).

Recently, we have noted a novel trend in the provision of private molecular genetic testing services, namely saliva and buccal swab collection kits (for DNA isolation) sold over the counter through pharmacies. These tests are very different from the standard pregnancy or blood sugar test kits that have long been sold over the counter in pharmacies since these latter kits do not involve DNA isolation or genetic analysis.

Several genetic testing laboratories in Europe and the USA appear to have entered into partnerships with pharmacies to provide their services to the public. These private genetic laboratories offer the following types of genetic test: (a) genetic tests for the detection of single-gene (Mendelian) disorders; (b) pharmacogenomic tests to individualize drug (including cancer) treatment, e.g., to guide specific mutation-targeted treatment decisions; (c) ‘predictive genomic testing’ for complex disorders and traits, e.g., heart/cardiovascular disease, osteoporosis, diabetes, athletic performance, etc.; (d) nutrigenomic tests, to individualize diet choices with the aim of bringing about weight loss; and (e) identity DNA testing. These tests may be organized into three main categories:

1. Genetic tests designed to diagnose inherited disorders and conditions that can be unambiguously assigned to specific genomic variants, namely (a) single-gene disorders and (b) drug-treatment efficacy or toxicity. For many of these tests, there is strong scientific evidence to support genotype-phenotype correlations, especially in the case of common genetic/genomic disorders. For a number of anticoagulant, psychiatric, and anti-cancer drugs, several pharmacogenomic markers have been identified in genes encoding drug-metabolizing enzymes and transporters that can predict drug efficacy or toxicity with a high degree of reliability, and many of them (including tests for variants in *CY2D6*, *CYP2C9*, *VKORC1*, and other pharmacogenes) have been cleared for use in clinical practice.

2. Genetic tests designed to diagnose complex health states and/or conditions characterized by a very poor or incomplete knowledge of genotype-phenotype correlations, e.g., cardiovascular disorders, diabetes, osteoporosis, etc., and/or with a very powerful gene-environment interaction, e.g., nutrient uptake, athletic performance, etc., that does not allow accurate estimation of disease risk based on an individual’s genetic profile. This situation is similar to attempting to solve a jigsaw puzzle that is missing several key pieces. In these genetic tests, risk profiles are calculated on the basis of risk markers with the aid of certain algorithms. When different genetic risk markers or algorithms are employed, different risk profiles may be generated, even for the same individual [9]! In this case, there is an omnipresent danger of obtaining a false-positive or false-negative result, which may either cause unnecessary alarm or alternatively provide inappropriate reassurance. Such misinterpretation of test results may in turn lead to the requisitioning of unwarranted, expensive, and potentially risky invasive diagnostic procedures or, conversely, could result in the individual concerned becoming less vigilant about their health.

3. Genetic tests that are ethically and/or legally questionable, despite being scientifically sound and accurate. This category of test is perhaps best exemplified by several well-publicized paternity cases in which the person whose DNA has been isolated and subsequently tested, has not provided the necessary consent (the so-called ‘infidelity DNA testing’). In these cases, the parties involved would be guilty of a misdemeanor punishable by law, at least in European countries and several US states [10].

This situation is somewhat different from the standard DTC genetic testing model since pharmacists are healthcare professionals who are in principle qualified to give appropriate advice to their clients, with the *proviso* of course that they have the necessary basic genetics training. In all these cases, and in several different countries, pharmacies are in effect acting as middlemen between the genetic testing providers and their customers.

There are certainly many benefits for the customers to be derived from the genetic tests which have been designed for the diagnosis of single-gene disorders and pharmacogenomics. First of all, by raising awareness of genetic issues, they can empower the individual to make decisions that could improve their health, including the adoption of better lifestyle choices. Moreover, the availability of this type of genetic test may contribute to a general decrease in the cost of genetic testing as a result of commercial competition. There are nevertheless a number of issues to be addressed in relation to selling DNA collection kits for genetic testing in pharmacies to ensure that the process is indeed beneficial to the patients involved and does not mislead the general public.

In the USA, regulatory clearance is required before a DNA collection kit (saliva, urine, or buccal swab tabs) can be commercially distributed. In 2010, the US Food and Drug Administration banned a commercial partnership between Walgreens, a retail pharmacy chain, and Pathway Genomics, a private genetic laboratory, from offering a variety of over-the-counter genetic tests since the company had not previously obtained regulatory clearance for the saliva collection kit to be sold as a medical device [11]. In contrast to the USA, such legislation is currently lacking in many European countries, even at a central level as a directive of the European Medicines Agency, as is the case for DTC genetic testing, where the environment is still not properly regulated [12].

Another major area of concern is the comparative lack of appropriate genetics education on the part of the healthcare professionals involved, namely pharmacists and physicians [13]. The genetic tests in question do not come with detailed pre- or post-test counseling, and so, the consumer is unlikely on their own to be able to interpret the test results satisfactorily or even appropriately. Further, most genetic test results come with a range of *caveats* and, if this is not fully appreciated by the user, misunderstanding of the clinical implications of these results is likely to be the rule rather than the exception. When physicians and pharmacists have been surveyed in the USA [14,15] and in some European countries [16,17], many have readily admitted that they lack the formal genetics education that would allow them to fully exploit the wealth of information that can now be obtained from genetic tests so as to deliver better healthcare to their patients. Further, they have acknowledged that even if the genetic test were potentially beneficial, they would not necessarily understand it well enough to be able to relay the appropriate diagnostic information to their patients. Unsurprisingly, both patients and the general public at large are often either unaware of the benefits of genetic testing or cannot easily distinguish between valid and informative genetic tests and those that will yield uninformative, unhelpful, or even potentially harmful results. As such, members of the public can easily be misled or end up being confused as to which test could be beneficial in their own particular case, and how effective that test could be at improving their long-term clinical outlook and/or decreasing their health costs. In addition, the general public could put themselves at risk if they (a) place undue reliance upon tests that have not been scientifically validated or approved by regulatory agencies and/or have intrinsic limitations based on existing scientific knowledge or (b) make important medical and lifestyle decisions based on these test results without first consulting an informed healthcare professional.

For all the reasons discussed above, we believe that some form of genetic counseling should ideally be integrated into, and made inseparable from, the genetic testing process [18]. To this end, we believe that genetic counseling should be made available to guide the patient through the entire genetic testing process (perhaps as a test-related single-session service) to determine what information an individual wishes to acquire from the test in question and what should be done with that information in terms of any post-test modification of their treatment regimen or lifestyle. Moreover, pharmacists should be obliged to receive some basic training about the genetic tests that they sell in their practices, at a bare minimum from the genetic laboratories that provide the tests, so that they are able to advise their clients accordingly and guide them to (and through) the test that is most appropriate to their needs. Pharmacists (and physicians) should also be encouraged to pursue continuous genetics education, in the form of, e.g., medical education-accredited seminars organized in concert with local universities and/or international organizations. This is particularly germane for those pharmacists who did not acquire the necessary level of genetics expertise during their undergraduate training. This notwithstanding, it should be pointed out that a recent survey in the USA indicated that very few participants utilized a genetic counseling service after direct-to-consumer personal genomic testing [19]. Ultimately, however, the decision as to whether or not have themselves avail of a genetic counseling service, offered at the point of sale of the genetic test, has to be the participant’s prerogative. Despite having some very real concerns as to how this might work in practice, we believe that with appropriate safeguards agreed and with the necessary legislation in place, it is likely that the current trend toward unregulated DTC testing will gradually decline of its own accord, and the partnerships between genetic testing laboratories and pharmacies will become better organized as they become better regulated. Patients and the general public alike should come to appreciate the fact that a genetic test is never going to be all enlightening, and that there will remain many limitations pertaining both to genetic testing and to the interpretation of genetic test results.

Finally, we must all understand that our knowledge of the genome remains at a fairly rudimentary stage which can, metaphorically speaking, be likened to the Phaestos disc [20], where one can just about understand the individual characters but certainly not the full meaning of the text.

## Competing interests

The authors declare that they have no competing interests.

## Authors’ contributions

GPP, DJB, FAM, VV, and DNC conceived and compiled the manuscript. All authors read and approved the final manuscript.
